# Massive Accumulation of Sphingomyelin Affects the Lysosomal and Mitochondria Compartments and Promotes Apoptosis in Niemann-Pick Disease Type A

**DOI:** 10.1007/s12031-022-02036-4

**Published:** 2022-06-21

**Authors:** Emma Veronica Carsana, Giulia Lunghi, Simona Prioni, Laura Mauri, Nicoletta Loberto, Alessandro Prinetti, Fabio Andrea Zucca, Rosaria Bassi, Sandro Sonnino, Elena Chiricozzi, Stefano Duga, Letizia Straniero, Rosanna Asselta, Giulia Soldà, Maura Samarani, Massimo Aureli

**Affiliations:** 1 Department of Medical Biotechnology and Translational Medicine, University of Milan, Milan, Italy; 2 Institute of Biomedical Technologies, National Research Council of Italy, Segrate, Milan, Italy; 3 Department of Biomedical Sciences, Humanitas University, Via Rita Levi Montalcini 4, 20090 Pieve Emanuele, Milan, Italy; 4 Humanitas Clinical and Research Center, IRCCS, Via Manzoni 56, 20072 Rozzano, Milan, Italy; 5 Department of Cell Biology and Infection, Institut Pasteur, Paris, France

**Keywords:** SMPD1, Sphingomyelin, Niemann-Pick, Lysosomes, Plasma membrane, Mitochondria

## Abstract

Niemann-Pick type A disease (NPA) is a rare lysosomal storage disorder caused by mutations in the gene coding for the lysosomal enzyme acid sphingomyelinase (ASM). ASM deficiency leads to the consequent accumulation of its uncatabolized substrate, the sphingolipid sphingomyelin (SM), causing severe progressive brain disease. To study the effect of the aberrant lysosomal accumulation of SM on cell homeostasis, we loaded skin fibroblasts derived from a NPA patient with exogenous SM to mimic the levels of accumulation characteristic of the pathological neurons. In SM-loaded NPA fibroblasts, we found the blockage of the autophagy flux and the impairment of the mitochondrial compartment paralleled by the altered transcription of several genes, mainly belonging to the electron transport chain machinery and to the cholesterol biosynthesis pathway. In addition, SM loading induces the nuclear translocation of the transcription factor EB that promotes the lysosomal biogenesis and exocytosis. Interestingly, we obtained similar biochemical findings in the brain of the NPA mouse model lacking ASM (ASMKO mouse) at the neurodegenerative stage. Our work provides a new in vitro model to study NPA etiopathology and suggests the existence of a pathogenic lysosome-plasma membrane axis that with an impairment in the mitochondrial activity is responsible for the cell death.

**Supplementary Information:**

The online version contains supplementary material available at 10.1007/s12031-022-02036-4.

## Introduction

Lysosomal storage disorders (LSDs) are characterized by the progressive accumulation of undigested macromolecules within the lysosomes due to loss of function mutations in genes coding for specific hydrolases or for proteins involved in the lysosomal function (Okada 1995). In particular, sphingolipidoses are LSDs characterized by defects in the catabolism of sphingolipids (SLs) leading to their accumulation. SLs are structural and functional lipids mainly localized at the plasma membrane (PM). Sphingomyelin (SM) is the most abundant SL and it is catabolized into ceramide and phosphocholine by the lysosomal enzyme acid sphingomyelinase (ASM; E.C. 3.1.4.12).

Nowadays, hundreds of mutations in the SMPD1 gene coding for ASM have been identified to cause SM accumulation with the onset of Niemann-Pick disease type A and B (NPA and NPB) (Schuchman and Desnick 2017). NPB is the late-onset form characterized by a higher residual ASM activity. Although NPB patients suffer from severe and progressive visceral organ abnormalities, they do not present symptoms involving the nervous system. On the other hand, NPA patients are characterized by a severe ASM deficiency (residual activity < 3% of that measured in healthy subjects) that leads to a rapid and progressive neurodegenerative course with fatal outcome in the first years after birth (Brady et al. 1966).

The pathogenic mechanism underlying NPA has been widely studied using both in vivo and in vitro models. In the 1990s, the availability of the SMPD1 knockout mouse model, known as ASMKO, opened a new scenario for the study of NPA. In particular, these mice showed a gradual accumulation of SM in the brain and the levels of the accumulated SM correlated with the severity of the pathology, indicating the necessity to overcome a threshold in order to evocate a neuronal damage (Horinouchi et al. 1995; Scandroglio et al. 2008). In addition, the alterations found in the brain of ASMKO mice suggest a progressive impairment of the endolysosomal compartment and of the autophagy flux that can be responsible for the secondary accumulation of other lipids such as gangliosides GM3 and GM2 (Buccinnà et al. 2009). Of note, the secondary accumulation of these gangliosides is a typical feature also found in other LSDs (Walkley and Vanier 2009; Pérez-Cañamás et al. 2017; Fuller and Futerman 2018). The first evidence associating the lysosomal accumulation of SM with the onset of neuronal damage was obtained by using in vitro cultures of hippocampal neurons from ASMKO mice. This study on ASMKO neurons revealed that the accumulation of SM is not only confined to lysosomes but also occurs at PM level (Gabandé-Rodríguez et al. 2014). Unfortunately, a limitation of this in vitro model is that these cells can be maintained in culture for a limited amount of time, not enough for reaching a sufficient level of SM accumulation comparable to that found in ASMKO mouse brain in neurodegenerative stages.

The findings obtained on the ASMKO mouse brain and in the derived neurons are in line with those found in an artificial in vitro model of LSDs, represented by human fibroblasts derived from a healthy donor and loaded with sucrose. Sucrose cannot be catabolized by fibroblasts because they lack the enzyme invertase; thus, it accumulates within lysosomes. The sucrose-loaded cells are characterized by a significant impairment of the lysosomal activity as well as of the autophagy flux that induces the nuclear translocation of the transcription factor EB (TFEB). TFEB is a master gene regulator of lysosomal genes, enhancing the lysosomal biogenesis and promoting lysosomal exocytosis. In particular, lysosomal exocytosis is responsible for alterations of the PM architecture (i.e., SL composition) that in turn lead to the onset of cell damage. These findings reveal the existence of a pathogenic lysosome-PM axis evocated by the aberrant accumulation of uncatabolized substrates in the lysosomes and shed new light on the study of LSDs (Gabandé-Rodríguez et al. 2014).

Here, we describe the development of a new in vitro model represented by fibroblasts isolated from an NPA patient, loaded for 30 days with exogenous SM in order to recapitulate the pathogenic phenotype, due to SM accumulation, observed in NPA patients. The molecular characterization of this model suggests that the SM accumulation activates a pathogenic lysosome-PM axis and impairs mitochondrial function, both events responsible for the onset of cell death.

## Materials and Methods

### Materials

Commercial chemicals were of the highest purity available, common solvents were distilled before use, and water was doubly distilled in a glass apparatus. Phosphate buffered saline (PBS) and calcium magnesium free (CMF)-PBS, glucose, sodium orthovanadate (Na_3_VO_4_), phenylmethanesulfonyl fluoride (PMSF), aprotinin, protease inhibitor cocktail, Triton X-100, bovine serum albumin (BSA), rabbit polyclonal anti-GAPDH antibody (RRID: G9535), polyclonal rabbit anti LC3IIB antibody (RRID: L7543), monoclonal mouse anti α-tubulin antibody (RRID: T5168), DNase I, trypsin, high performance thin layer chromatography (HPTLC) plate, NaCl, KCl, NaF, EDTA, trizma base, glycerol, MgCl_2_, sodium dodecyl sulfate (SDS), paraformaldehyde, digitonin, and RPMI-1640 were from Sigma-Aldrich (St. Louis, MO, USA). L-Glutamine, penicillin/streptomycin (10,000 U/ml/10 mg/ml), fetal bovine serum (FBS), and EuroGoldTriFast reagent were from EuroClone (Paignton, UK). Lysenin antiserum was from Peptides International (Louisville, KY, USA). LysoTracker Red DND-99 was from Molecular Probes (Eugene, OR, USA). MitoSOX™ Red Mitochondrial Superoxide Indicator, MitoTracker™ Deep Red FM, Calcein-AM, DMEM-F12, HEPES, polyclonal anti-rabbit AlexaFluor488 (RRID: A11008), and anti-mouse AlexaFluor568 (RRID: A10037) were from ThermoFisher (Waltham, MA, USA). Polyclonal anti-Cleaved Caspase-3 (Asp175) antibody (RRID:9661), monoclonal anti-HtrA2/Omi (D20A5) antibody, monoclonal anti-p38 MAPK (D13E1), monoclonal anti-phospho-p38 MAPK (Thr180/Tyr182) (28B10), and anti-Histone H3 (RRID: 4499) were from Cell Signaling Technology, Danvers, MA, USA. Monoclonal anti-PINK1 (38CT20.8.5) and monoclonal anti-Parkin antibody were from Santa Cruz Biotechnology (Dallas, TX, USA) Sphingomyelin was from Avanti Polar Lipids (Alabaster, AL, USA). Rabbit anti-TFEB (RRID: 7942) was from Bethyl Laboratories (Montgomery, TX, USA). Complete Protease Inhibitor Cocktail Tablets were from Roche (Basilea, Switzerland).

Monoclonal anti-Mitofusin 2 + Mitofusin 1 (3C9) antibody was from Enzo Life Sciences (Farmingdale, New York, USA). Monoclonal mouse anti-LAMP1 antibody (H4A3) was from Developmental Studies Hybridoma Bank (Iowa City, IA, USA); polyclonal anti-TOM20 antibody (RRID: 11,802–1-AP) was from Proteintech (Manchester, UK); total Oxphos Human WB Antibody Cocktail (RRID: ab110411) and monoclonal anti-Mitofusin 2 + Mitofusin 1 (3C9) antibody were from Abcam (Cambridge, UK). Mouse monoclonal anti-calnexin antibody (RRID: 610,524) was from BD Franklin Lakes (Bergen, NJ USA). The chemiluminescence kit for immunoblotting was from Cyanagen (Bologna, Italy). ATP detection assay system and Liquid scintillation cocktail Ultima Gold were from Perkin Elmer (Waltham, MA, USA). A total of 4–20% Mini-PROTEAN® TGX™ Precast Protein Gels, Turbo Polyvinylidene difluoride (PVDF) Mini-Midi membrane, DC™ protein assay kit, and DTT were from BioRad (Hercules, CA, USA). 4-Methylumbelliferyl-b-D-galactopyranoside (MUB-Gal), 4-methylumbelliferyl-b-D-glucopyranoside (MUB-Glc), 4-Methylumbelliferyl-β-D-glucosaminide (MUG), and 4-methylumbelliferone were from Glycosynth (Warrington, UK); Illumina TruSeq Stranded mRNA Sample LS Preparation Kit was from Illumina (San Diego, CA, USA).

### Cell Cultures

Fibroblasts were obtained by skin biopsy of a healthy subject or a patient affected by NPA. Healthy and NPA fibroblasts were derived from the “Cell line and DNA Biobank from patients affected by Genetic Diseases” of the Istituto G. Gaslini (Genova, Italy) after written donor informed consent, approved by the local Ethics Committee.

Fibroblasts were cultured in RPMI-1640 medium supplemented with 10% heat-inactivated FBS, 2 mM L-glutamine, 100 U/ml penicillin, and 100 µg/ml streptomycin. Cells were cultured as monolayer in a humidified atmosphere at 37 °C, 5% CO_2_.

### Preparation of Mouse Brain Tissue Homogenates

The starting acid sphingomyelinase knockout (ASMKO) mouse model was produced in the E.H. Schuchman’s laboratory (Department of Genetics and Genomic Sciences, Mount Sinai School of Medicine, New York, NY, USA) and then backcrossed onto the C57BL/6 N strain at the Charles River Laboratory, Milan, Italy. For the experiments reported in this work, wild type (WT) mice (C57BL/6 N; ASM^+/+^) were bred to each other, as were the mutant mice (C57BL/6 N; ASM^−/−^) to obtain homozygous offspring.

All animal procedures were approved by the Ethics Committee of the University of Milano, Italy, and were performed in accordance with the National Institute of Health Guide for the Care and Use of Laboratory Animals (Directive 2010/63/EU) (project numbers 13–2015-PR, 14–2015-PR, and 15–2015-PR).

Wildtype (WT) and acid sphingomyelinase knockout (ASMKO) mice were sacrificed at 7 months after birth and brains were collected and weighed.

#### Brain Tissue Homogenates for Western Blot Analysis

After mechanical homogenization in ice, brain tissue was resuspended in lysis buffer (10 mM HEPES at pH 7.9, 1.5 mM MgCl_2_, 10 mM KCl, 0.2 mM EDTA) supplemented with 0.32 M sucrose, 1 mM DTT, 1 mM NaF, 1 mM, Na_3_VO_4_, and Protease Inhibitor Cocktail with a ratio of 0.1 g fresh tissue/0.9 ml lysis buffer. Brain tissue was then homogenized with a Teflon pestle in a Potter–Elvehjem tissue grinder and centrifuged at 850 × g for 10 min at 4 °C. The supernatant was removed and the pellet was resuspended in lysis buffer supplemented with 1 mM DTT and Protease Inhibitor Cocktail. The protein concentration of tissue homogenates was determined by DC Protein Assay. Aliquots of tissue homogenates were further processed for nuclear extraction.

#### Brain Tissue Homogenates for Enzymatic Activities

After washing in PBS, brains were mechanically homogenized and then resuspended in 10 volumes of McIlvaine buffer (0.1 acid citric/0.2 Na_2_HPO_4_) at pH 6 containing Complete Protease Inhibitor Cocktail Tablets and PMSF 1 mM. After homogenization in a Potter–Elvehjem homogenizer at 4 °C, homogenates were sonicated followed by centrifugation at 300 × g for 10 min at 4 °C. Supernatants were then collected for determination of protein concentration by DC Protein Assay.

### Sphingomyelin Loading

NPA fibroblasts were plated at a density of 3000 cells/cm^2^ and cultured for 30 days in complete growth medium supplemented or not with 50 µM SM. An aliquot corresponding to 5 µmol of SM was taken from a stock solution (25 mg/ml in chloroform:methanol 2:1), transferred in a sterile tube, dried under a nitrogen flow, and solubilized in 10 ml of heat-inactivated FBS supplemented with antibiotics to obtain 500 µM final concentration. After stirring and sonication using ultrasonic bath (1 min, three times), the solution was incubated at 37 °C overnight, diluted 1:10 in the culture medium, and added to the cells 24 h after plating. Control NPA fibroblasts were cultured in the same conditions without SM. Culture media were changed every 7 days and SM was re-administered every week for 1 month.

### Treatment of Cell Cultures with [.^3^H]-SM

Isotopically labeled [3-^3^H(sphingosine)]-SM (specific radioactivity 0.375 Ci/mmol) was prepared by feeding healthy fibroblasts with [3-^3^H]sphingosine and purified from total lipid extract by flash chromatography (Toyokuni et al. 1991). [3-^3^H(sphingosine)]-SM dissolved in propanol: H_2_O 7:3 (v:v) was transferred into a sterile glass tube, dried under a nitrogen stream, solubilized in an appropriate volume of FBS (0.04 µCi /ml) as described for the SM loading, and administered to cells.

### Protein Determination

Protein concentration of samples was assessed by DC™ protein assay kit according to manufacturer’s instructions, using BSA at different concentrations as standard.

### Immunoblotting

Immunoblotting analysis of homogenates from WT and ASMKO mouse brains and lysates from NPA fibroblasts loaded or not with SM was performed using standard protocols. Aliquots of proteins were mixed with Laemmli buffer (0.15 M DTT, 94 mM Tris–HCl, 15% glycerol, 3% SDS w: v, 0.015% blue bromophenol, v: v) and heated for 5 min at 95 °C. For each sample, the same amount of proteins was separated on polyacrylamide gels and transferred to PVDF membranes by electroblotting. PVDF membranes were incubated in blocking solution with 5% non-fat dry milk in TBS-0.1% tween-20 at 23 °C for 1 h under gentle shaking. Subsequently, PVDF membranes were incubated overnight at 4 °C with primary antibodies diluted in blocking solution. PVDF membranes were then incubated 1 h at 23 °C with secondary HRP-conjugated antibodies diluted in blocking solution. PVDF were scanned using the chemiluminescent system Alliance Mini HD9 (Uvitec, Cambridge, UK) and band intensity was quantified using the ImageJ software (v2.1.0/1.53c; NIH, Bethesda, USA; http://rsbweb.nih.gov/ij). The following primary antibodies diluted in blocking solution were used for immunoblotting: monoclonal mouse anti-LAMP1 (1:100); polyclonal rabbit anti-GAPDH (1:7000); polyclonal rabbit anti LC3IIB antibody (1:1000); polyclonal anti-Cleaved Caspase-3 (Asp175) antibody (1:1000); polyclonal anti-TOM20 antibody (1:1000); mouse anti-total Oxphos Antibody Cocktail (1:5000 in 3% BSA in TBS 0.1% tween-20); monoclonal mouse anti α-tubulin antibody (1:20,000); mouse monoclonal anti-calnexin antibody (1:1000 in 5% BSA in TBS 0.1% tween-20); mouse monoclonal anti-Mitofusin 2 + Mitofusin 1 (3C9) antibody (1:1000); rabbit polyclonal anti Fis1 antibody (1:1000); mouse monoclonal anti-Parkin antibody (1:500); rabbit monoclonal anti-HtrA2/Omi (D20A5) antibody (1:1000); mouse monoclonal anti-PINK1 (38CT20.8.5) (1:500); rabbit monoclonal anti-p38 MAPK (D13E1) (1:1000); and mouse monoclonal anti-phospho-p38 MAPK (Thr180/Tyr182) (28B10) (1:1000 in 5% BSA in TBS 0.1% tween-20). The following secondary antibodies diluted in blocking solution were used: Goat-anti-rabbit HRP-conjugated (RRID: 7074; 1: 10,000) and Goat-anti-mouse HRP conjugated (RRID: 31,430; 1:2000).

### Evaluation of Enzymatic Activities in Cell Lysates and Tissue Homogenates

The enzymatic activities were determined in total cell lysates and brain homogenates as previously reported (Aureli et al. 2011) using 4-Methylumbelliferone (MUB)-derived fluorogenic substrates MUB-β-Glc, MUB-β-Gal, and MUG.

To assay β-glucocerebrosidase activity, aliquots of cell lysates or tissue corresponding to 20 µg of proteins were pre-incubated for 30 min at 23 °C in a 96-well microplate in a reaction mixture composed of 25 µl of McIlvaine buffer 4X (0.4 M citric acid, 0.8 M Na_2_HPO4) pH 5.2; 5 nM AMP-DNM (Adamantane-pentyl-dNM;N-(5-adamantane-1-yl-methoxy-pentyl)-Deoxynojirimycin), the selective inhibitor of the non-lysosomal β-glucosylceramidase; and water to a final volume of 75 µl. The reaction mixtures were incubated at 37 °C under gentle shaking. At the end of pre-incubation, the reaction was started adding 25 µl of MUB-β-Glc at a final concentration of 6 mM.

To assay β-galactosidase and β-hexosaminidase activities, 5 µg of proteins was incubated at 37 °C under gentle shaking in a 96-well microplate with 25 µl of McIlvaine buffer 4X pH 5.2 containing the specific fluorogenic substrates MUB-β-Gal and MUG at a final concentration of 500 µM and water was added to a final volume of 100 µl. At different time points, 10 µl of the reaction mixtures was transferred to a black microplate (Black, 96-well, OptiPlate-96 F, Perkin Elmer) and 190 µl of 0.25 M glycine pH 10.7 was added. The MUB-associated fluorescence was detected by a microplate reader (Victor, Perkin Elmer) (ex/em 365/460 nm). Data were expressed as nanomoles of converted substrate/hour/milligrams of proteins.

### Evaluation of Plasma Membrane Enzymatic Activities in Living Cells

The activity of β-glucocerebrosidase, β -galactosidase, and β-hexosaminidase at the plasma membrane level was assayed using a high-throughput cell lived-based assay as previously described (Aureli et al. 2011). Cells were plated in 96-well microplate at a density of 3000 cells/cm^2^; 24 h after plating, cell culture medium was removed and cells were rinsed with DMEM-F12 without phenol red. Artificial substrates (MUB-Gal, MUG), solubilized in DMEM-F12 without phenol red at pH 6 at a final concentration of 1 mM and 6 mM, respectively, were added to the cells. For β-glucocerebrosidase assay, cells were pre-incubated for 30 min at room temperature with 5 nM AMP-DNM in DMEM/F-12. After 2 h incubation at 37 °C, 10 µl of the reaction mixtures was transferred to a black microplate (Black, 96-well, OptiPlate-96 F, Perkin Elmer) and 190 µl of 0.25 M glycine pH 10.7 was added. The MUB-associated fluorescence was detected by the Victor microplate reader (ex/em 365/460 nm). Since fluorogenic substrates are not able to cross the plasma membrane, under these experimental conditions, the measured fluorescence is exclusively attributable to the hydrolytic activity of PM glycohydrolases. Enzymatic activity was expressed as nanomoles of product/hour/10^6^ cells.

### Analysis of Radioactive Lipid Pattern by HPTLC

After 30 days in culture, cells were harvested and lysed with H_2_O supplemented with protease and phosphatase inhibitors and the cell lysates were then lyophilised. Total lipids were extracted from lyophilized cell lysates with chloroform:methanol:water (2:1:0.1, v:v:v) and separated from the pellet by centrifugation at 13,000 × g for 15 min, followed by a second and third extraction with chloroform:methanol, 2:1 by vol. The radioactivity associated with the total lipids (TL) was determined by liquid scintillation counting by beta-counter (PerkinElmer). Lipids were resolved by monodimensional HPTLC using the solvent system: chloroform:methanol:water (110:40:6, v:v:v). Lipids were identified by comigration with authentic standards such as ceramide, glucosylceramide, globotriaosylceramide, phosphatidylethanolamine, SM, and ganglioside GM3 which were available in laboratory (Mauri et al. 2018). Radioactive lipids were detected by radioactivity imaging (Beta-Imager ^T^Racer Betaimager, BioSpace Laboratory, Nesles-la-Vallée, France) and quantified using M3 Vision software (Biospace Lab Inc.).

### Analysis of the Endogenous Lipid Pattern by HPTLC

Total lipids (TL) were extracted and partitioned as previously described in order to obtain an aqueous phase (AP) mainly containing gangliosides and an organic phase (OP) mainly containing neutral glycolipids, phospholipids, and apolar lipids. Lipids were separated using different solvent systems: chloroform:methanol:CaCl_2_ (50:42:11, v:v:v) for ganglioside analysis of AP; chloroform:methanol:water (110:40:6, v:v:v) for neutral glycolipids of the OP; hexane:ethylacetate (3:2, v:v) for cholesterol of TL; chloroform:methanol:acetic acid:water (30:20:2:2, v:v:v:v) for phospholipids analysis of the OP. After lipid separation, HPTLC was revealed using different spraying reagents: the anysaldehyde reagent was used to reveal neutral lipids and cholesterol; the Ehrlich reagent was used to visualize gangliosides; and the phosphorus reagent was used to detect phospholipids. Lipids were identified by comigration with authentic standards such as ceramide, glucosylceramide, lactosylceramide, globotriaosylceramide, phosphatidylcholine, phosphatidylethanolamine, SM, cholesterol, gangliosides GM3, GM2, GD3, and GD1a which were available in laboratory (Mauri et al. 2018). HPTLC was scanned and band intensity quantified using the ImageJ software. For each sample, the relative quantitative analyses of SM were performed interpolating the mean value of each band from a calibration curve obtained with SM standards at known concentration separated and quantified on the same HPTLC plate.

### Lysotracker Staining

NPA fibroblasts were plated in 6-well plates at a density of 3000 cells/cm^2^ and were loaded or not with SM once a week for 30 days. LysoTracker Red DND-99 was directly diluted in the culture medium at 50 nM final concentration (30 min, 37 °C, 5% CO_2_). Cells were washed with PBS and images were acquired with Olympus IX50 Inverted Fluorescence Microscope equipped with a halogen lamp directly on live cells at 200 × magnification.

### Lysenin Staining

Cells plated on glass coverslips were permeabilized with 50 µg/ml digitonin in PBS for 10 min at RT. Cells were then washed three times with PBS and incubated in blocking solution (2% BSA in PBS) for 15 min at RT. After three washings with PBS, cells were incubated with 1 µg/ml lysenin in blocking for 2 h at RT. Cells were then washed three times with PBS and incubated with rabbit lysenin antiserum (1:500 in blocking solution, 1 h at RT). Cells were washed three times with PBS and incubated with anti-rabbit AlexaFluor488 antibody (1:600 in blocking solution, 45 min at RT). After PBS washes, coverslips were mounted on a glass slide with Dako fluorescent mounting medium. The fluorescence emitted by Lysenin was detected using a NikonEclipse Ni upright microscope (400 × magnification) and a Nikon DIGITAL SIGHT DS-U1 CCD camera. Images were analyzed and fluorescence intensity was quantified using the ImageJ software. At least 20 fields were examined for each sample.

### MitoTracker™ Deep Red FM Staining

NPA Fibroblasts were plated at a density of 3000 cells/cm^2^ in 6-well plates on glass coverslips and were loaded or not with SM once a week. After 30 days, cells were incubated with 125 nM MitoTracker™ Deep Red FM in complete medium for 20 min. Cells were washed three times with cold PBS and fixed with 4% (v/v) paraformaldehyde in PBS (20 min, 23 °C). Cells were stained with 0.0002% (v/v) Hoechst in PBS (5 min, 23 °C), washed with PBS, and mounted with Dako fluorescent mounting medium. The fluorescence emitted by MitoTracker™ Deep Red FM was detected using a NikonEclipse Ni upright microscope (400 × magnification) and a Nikon DIGITAL SIGHT DS-U1 CCD camera. Images were analyzed and fluorescence intensity was quantified using the ImageJ software (at least 20 fields were examined for each sample).

### Evaluation of Mitochondrial Superoxide Generation

The production of mitochondrial superoxide was performed with MitoSOX Red. NPA Fibroblasts were plated at a density of 3000 cells/cm^2^ in 6-well plates on glass coverslips and were loaded or not with SM once a week. After 30 days, cells were incubated with 2 μM MitoSOX (10 min, 37 °C). Cells were washed three times with cold PBS and fixed with 4% (v/v) paraformaldehyde in PBS (20 min, 23 °C). Cells were stained with 0.0002% (v/v) Hoechst in PBS (5 min, 23 °C) and mounted with Dako fluorescent mounting medium. The fluorescence emitted by MitoSOX Red was detected using a NikonEclipse Ni upright microscope (400 × magnification) and a Nikon DIGITAL SIGHT DS-U1 CCD camera. Images were analyzed and fluorescence intensity was quantified using ImageJ software. At least 20 fields were examined for each sample and images analyzed as specified above.

### Total ATP Assay

Cells were plated in 96-well microplate at a density of 3000 cells/cm^2^ and loaded or not with SM once a week for 30 days. Total ATP production was measured using the luminescence ATP detection assay system ATPlite kit. Luminescence was detected by a microplate reader and total ATP production was quantified and normalized to cell viability evaluated as fluorescence signal by calcein-AM assay as described in the next paragraph.

### Calcein-AM Cell Viability Assay

The nonfluorescent Calcein AM is a cell-permeant dye that in live cells is converted to a green fluorescent calcein after hydrolysis by intracellular esterases. Cells were plated in 96-well microplate at a density of 3000 cells/cm^2^ and loaded or not for 30 days with SM. After culture medium removal, cells were washed with PBS and then incubated with 5 µg/ml Calcein AM solution in PBS (15 min, 37 °C). At the end of incubation, Calcein-AM solution was discarded and cells were lysed with 1% Triton X-100 in TNEV buffer (10 mM TrisHCl pH 10, 150 mM NaCl, 5 mM EDTA pH 7.5) in mild agitation (10 min, 23 °C). The cell lysates were transferred to a black microplate (Black, 96-well, OptiPlate-96 F, Perkin Elmer) and fluorescence intensity (ex/em 495/515 nm) was measured by the Victor microplate reader.

### Nuclear Purification from Cells

Nuclear extraction was performed as previously described (Settembre and Medina 2015). Briefly, cells were lysed with 0.5 ml of lysis buffer (50 mM Tris–HCl (pH 7.5), 0.5% Triton X-100, 137.5 mM NaCl, 10% glycerol, and 5 mM EDTA) supplemented with Protease Inhibitor Cocktail and 1 mM Na3VO4 (15 min in ice under gentle shaking). Lysates were centrifuged at 15,700 g for 15 min at 4 °C. The nuclear pellet was rinsed 3 times with 0.5 ml of lysis buffer, resuspended in 0.1 ml of lysis buffer supplemented with 0.5% SDS, and sonicated in ice. Nuclear extract was collected after centrifugation at 15,700 g for 15 min at 4 °C.

### Nuclear Purification from Mouse Brain Tissue

Brain tissue homogenates were centrifugated at 850 × g for 10 min at 4 °C. Cell pellet was resuspended in extraction buffer (20 mM HEPES at pH 7.9, 1.5 mM MgCl_2_, 0.42 M NaCl, 0.2 mM EDTA, 25% v/v glycerol) supplemented with 1 mM DTT and Protease Inhibitor Cocktail. After Dounce homogenization (10 hits with tight pestle and 10 hits with loose pestle), the suspension was maintained in gentle shaking for 45 min at 4 °C and centrifuged at 20,000 × g (20 min at 4 °C). The supernatant, corresponding to the nuclear extract, was dialyzed against 50 volumes of dialysis buffer (20 mM HEPES, pH 7.9, 1.5 mM MgCl_2_, 10 mM KCl, 0.2 mM EDTA, 20% v/v glycerol) supplemented with 1 mM DTT and Protease Inhibitor Cocktail for 24 h at 4 °C.

### Electron Microscopy of Cell Monolayers

Cells monolayers were fixed in a mixture of 4% paraformaldehyde and 2% glutaraldehyde in cacodylate buffer (0.12 M, pH 7.4) for 4 h at 4 °C. Then, cells were extensively washed with cacodylate buffer and subsequently post-fixed for 1 h on ice in a mixture of 1% osmium tetroxide and 1.5% potassium ferrocyanide in cacodylate buffer. After several washes with ultrapure water, samples were “en bloc” stained with 0.5% uranyl acetate in water overnight at 4 °C. Finally, samples were dehydrated in a graded ethanol series and then infiltrated for 2 h in a mixture 1:1 (v/v) of ethanol and Epon and subsequently in 100% Epon, twice for 1 h. Then polymerization was performed for 24 h in an oven at 60 °C. Ultra-thin Sects. (80 nm) were prepared using an ultramicrotome (Leica Ultracut; Leica Microsystems GmBH, Wien, Austria), collected on nickel grids and stained with saturated uranyl acetate for 5 min and washed and then stained with 3 mM lead citrate for 5 min. Finally, the sections were photographed using a transmission electron microscope LEO 912AB (Advanced Light and Electron Microscopy BioImaging Center—San Raffaele Scientific Institute, Milano, Italy).

### RNA Analysis

RNAseq was performed on NPA fibroblasts cultured in presence or absence of SM for 30 days. For each condition (loaded or unloaded), the experiments were performed in triplicate, for a total of 6 samples.

Total RNA was extracted using the EuroGoldTriFast reagent according to the manufacturer’s instructions. RNA concentration was determined using a NanoDrop ND-1000 Spectrophotometer (Thermo Fisher Scientific, Waltham, MA, USA) and RNA integrity was assessed on a LabChip GX Touch (PerkinElmer), obtaining a mean RNA integrity number (RIN) of 9.1 (max: 9.4, min: 8.5). For each sample, 500 ng of RNA was used to generate paired-end sequencing libraries with an Illumina TruSeq Stranded mRNA Sample LS Preparation Kit, according to the manufacturer’s protocol. Sequencing was performed on a NextSeq500 platform (Illumina) at the Humanitas Genomic Facility (Milan, Italy). Reads were mapped to the hg38 reference genome and to the UCSC *Homo sapiens* transcriptome (Illumina’s iGenomes reference annotation, downloaded from University of California, Cruz (UCSC); http://support.illumina.com/sequencing/sequencing_software/igenome.html) with STAR v.2.7.1a (Dobin et al. 2013) and gene counts were generated with htseq-count v.0.11.2 using the following parameters: -m intersection-nonempty -t exon -s reverse. Quality control (QC) report was generated with multiQC v.1.7 including QC statistics from FastQC v.0.11.8, STAR, htseq-count, and from the CollectRnaSeqMetrics tool from Picard v.2.20.8 (http://broadinstitute.github.io/picard/). Differential expression analysis between treated and untreated samples (design: replicate + treatment) was evaluated with the Wald χ^2^ test of significance for the negative binomially distributed counts using the DeSeq2 Bioconductor package (Love et al. 2014), after removing genes with extremely low counts (less than 10 counts overall). Differentially expressed (DE) genes were selected using a False Discovery Rate (FDR) of ≤ 0.01 and a fold change of ≥ 2 or ≤ − 2. Gene ontology (GO) enrichment analyses were performed using Metascape (Zhou et al. 2019) with default parameters, separately on upregulated and downregulated genes. Expression profiling data were submitted to the Gene Expression Omnibus repository (Accession Number: GSE199194).

### Statistical Analyses

All statistical analyses were performed using GraphPad Prism 7 (GraphPad Software Inc., La Jolla, CA, USA). Normal distribution was tested by D’Agostino-Pearson test and/or Shapiro–Wilk test. Data are expressed as mean ± SEM. For normally distributed data, two-sided unpaired Student’s *t* test was used. A *p*-value < 0.05 was considered significant.

## Results

### Development of an In Vitro Model for the Study of the NPA Disease

Fibroblasts derived from NPA patients represent an easy-to-use in vitro model for the study of NPA, since they recapitulate the ASM deficiency. In our study, we used fibroblasts derived from a healthy subject and those derived from an NPA patient, which are characterized by an ASM residual activity of 1.3% with respect to the healthy ones (data not shown).

**Fig. 1:**
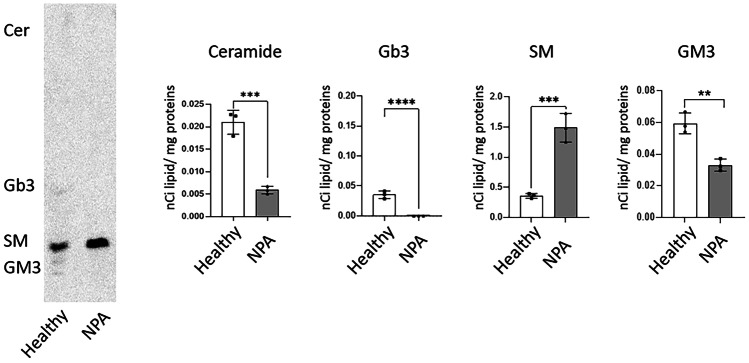
Metabolic fate of radioactive sphingomyelin in fibroblasts of a patient affected by Niemann-Pick type A disease. Representative HPTLC image of sphingolipid pattern and quantification of the radioactivity associated with ceramide (Cer), globotriaosylceramide (Gb3), sphingomyelin (SM), and ganglioside GM3 of fibroblasts of a healthy and a Niemann-Pick type A (NPA) patient. Cells were cultured for 30 days and fed with [.^3^H]-SM for other 7 days. Data are expressed as nCi of lipid/mg proteins and are the mean ± SEM of three different experiments. **p < 0.01, ***p < 0.001, ****p < 0.0001 two-tailed Student’s *t*-test vs healthy fibroblasts

To verify the impairment in the SM catabolism, we fed both healthy and NPA fibroblasts with radioactive [^3^H-Sph]-SM for 7 days and performed lipid extraction and HPTLC separation. As shown in Fig. 1, NPA fibroblasts were characterized by a fourfold higher SM content compared to healthy fibroblasts. Moreover, NPA fibroblasts did not show any other catabolite such as ceramide and GM3 or any metabolite derived from the salvage pathway, such as globotriaosylceramide (Gb3), with respect to healthy cells. Conversely, both cell lines were characterized by the same proliferation rate and viability (data not shown), thus indicating that SM accumulation does not reach a threshold leading to cell damage.

**Fig. 2:**
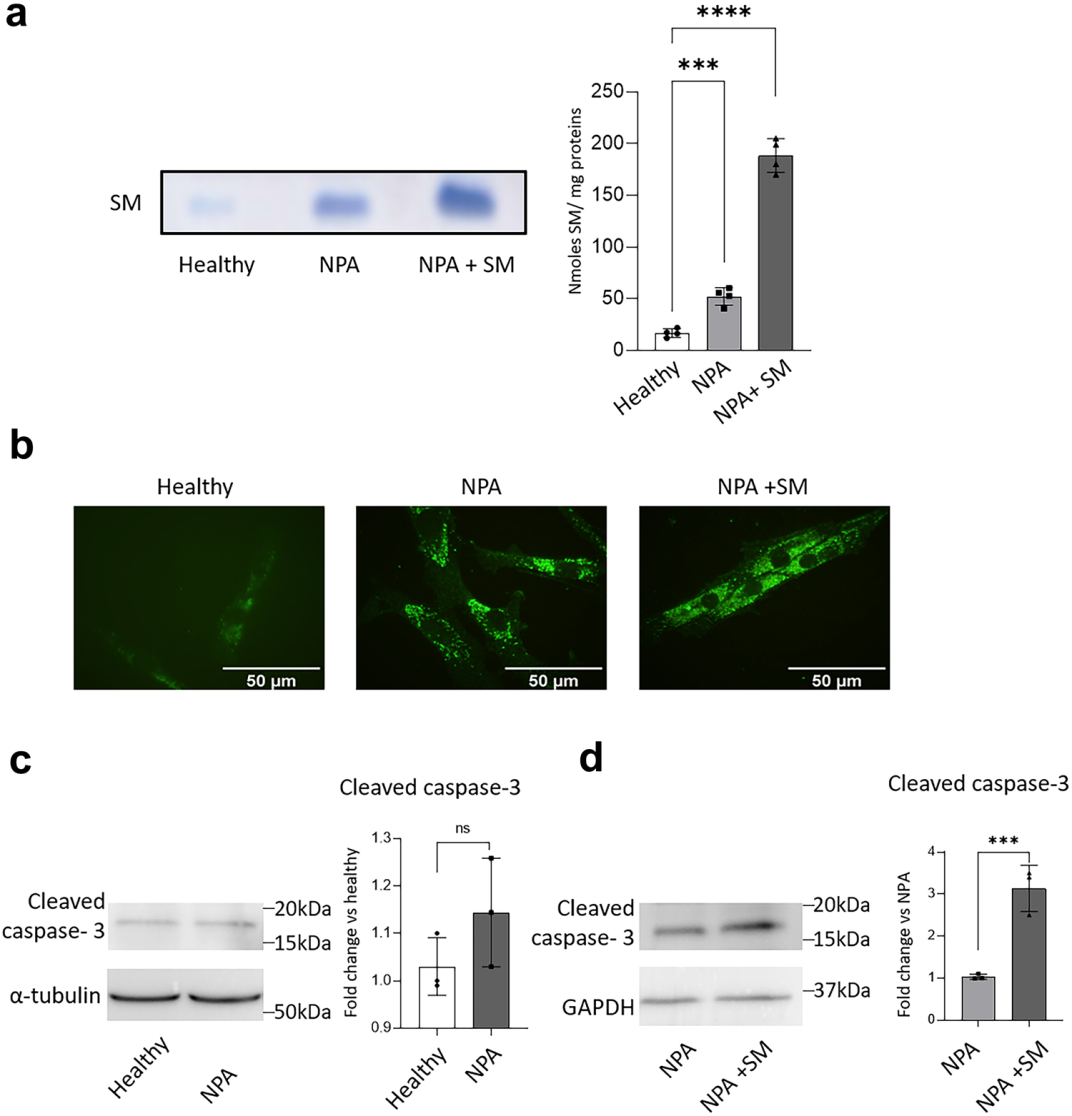
Effect of SM loading on its accumulation and cell death in Niemann-Pick type A fibroblasts. **a** Representative HPTLC separation of sphingomyelin (SM) of healthy and NPA fibroblasts loaded or not with 50 µM SM for 30 days. SM content was quantified by densitometric analysis; data are expressed as nmoles of SM/mg of cell proteins and are the mean ± SEM of three different experiments. **p < 0.01; ****p < 0.0001; two-tailed Student’s *t*-test vs healthy fibroblasts. **b** Representative images of the SM staining with lysenin in healthy fibroblasts and NPA fibroblasts loaded or not with 50 µM SM for 30 days. Images were acquired at 200 × magnification. **c** Immunoblotting analysis of the expression of cleaved caspase-3 in healthy and NPA fibroblasts. **d** Immunoblotting analysis of the expression of cleaved caspase-3 in NPA cells loaded or not with 50 µM SM for 30 days and healthy fibroblasts. Optical densities of the individual bands were quantified using NIH ImageJ and normalized to α-tubulin or GAPDH. Data are expressed as fold change with respect to healthy cells (**c**) or unloaded NPA cells (**d**) and are the mean ± SEM of three different experiments. Ns = nonsignificant; ***p < 0.001; two-tailed Student’s *t*-test vs unloaded NPA cells

Based on these results, we developed a new cell model able to recapitulate a massive accumulation of SM in vitro. Briefly, NPA fibroblasts were fed for 30 days with 50 µM exogenous SM, a concentration approximately eightfold higher than the one measured in FBS used for the cell culture (Chigorno et al. 2005). At the end of the treatment, we analyzed the SM content of NPA fibroblasts, loaded or not with exogenous SM and healthy fibroblasts. As shown in Fig. 2a, the higher SM content of NPA fibroblasts (fourfold increase vs healthy fibroblasts) further augmented in SM-loaded NPA fibroblasts reaching an amount of 177.5 nmol/mg ± 10.27 (tenfold increase vs healthy fibroblasts). We also evaluated the intracellular accumulation of SM by indirect immunofluorescence analysis of cells labeled with lysenin, a toxin that selectively binds SM. As shown in Fig. 2b, a more marked lysenin staining was observable in NPA fibroblasts with respect to healthy ones, which was even higher and diffused throughout cytoplasm upon SM loading. Notably, we observed a prominent perinuclear SM localization likely suggesting its lysosomal accumulation.

Moreover, in SM-loaded NPA cells, we found the activation of the apoptotic pathway, as demonstrated by the 2.5-fold increase in the levels of the effector caspase-3 (Fig. 2d).

These data indicate that the continuous administration of exogenous SM to NPA fibroblasts induces a further SM accumulation leading to the onset of cell death.

### The Aberrant Accumulation of SM is Paralleled by the Secondary Accumulation of Other Lipids

**Fig. 3:**
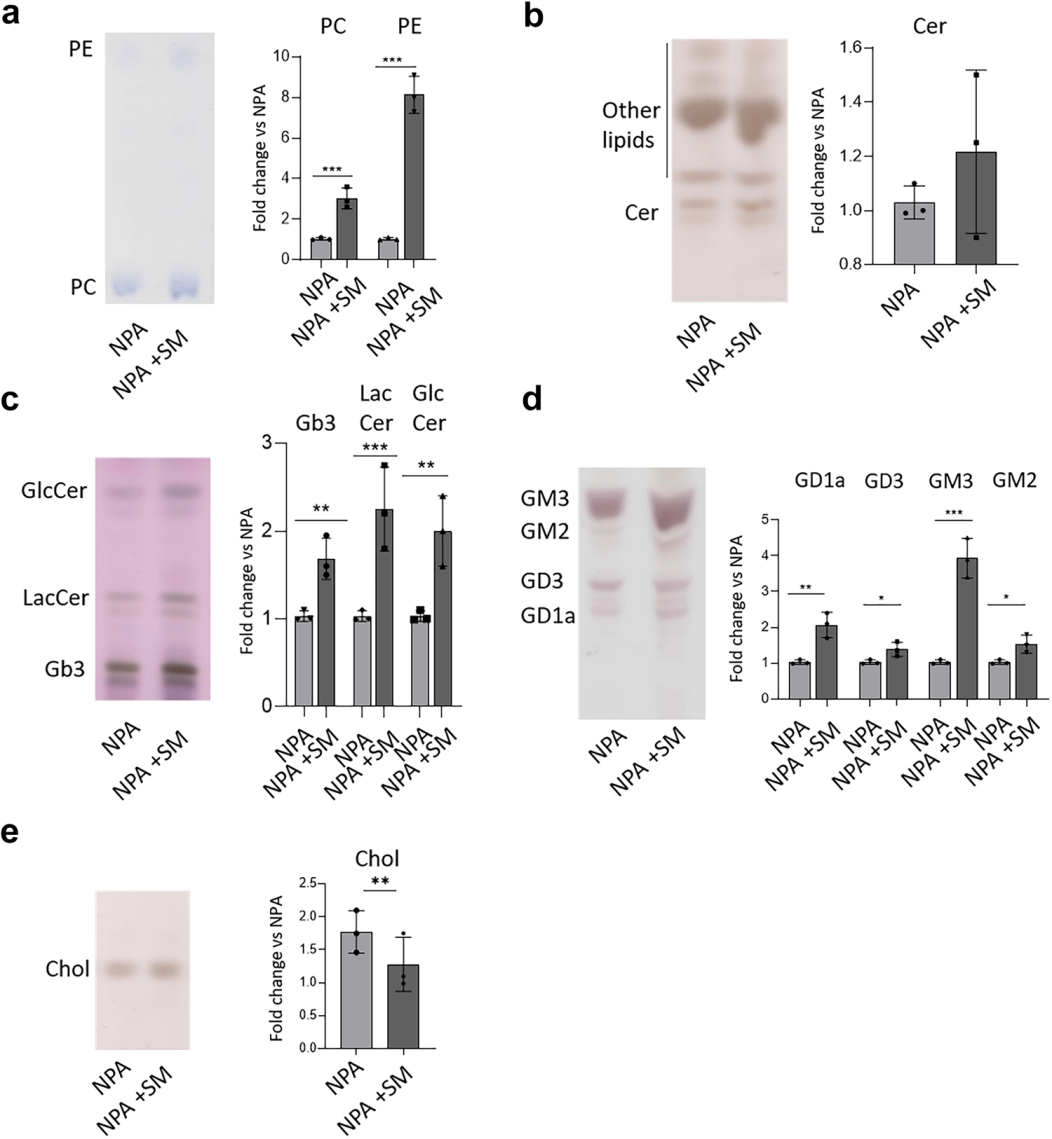
Effect of sphingomyelin accumulation on the lipid pattern. Representative HPTLC separations of endogenous lipids: **a** phospholipids, **b** ceramide, **c** neutral lipids, **d** ganglioside pattern, and **e** cholesterol of NPA fibroblasts loaded or not with 50 µM SM. PE = phosphatidylethanolamine; PC = phosphatidylchline; Chol = cholesterol; Cer = ceramide; GlcCer = glucosylceramide; LacCer = lactosylceramide; Gb3 = globotriaosylceramide; GD1a = Disialoganglioside GD1a; GD3 = Disialoganglioside GD3; GM3 = monosialogangloside GM3; GM2 = monosialogangloside GM2. Lipid content was quantified by densitometric analysis; data are expressed as fold change with respect to NPA fibroblasts and are the mean ± SEM of three different experiments. *p < 0.05; **p < 0.01; ***p < 0.001, ****p < 0.0001; two-tailed Student’s *t*-test vs unloaded NPA cells

A typical feature of LSDs is the secondary accumulation of lipids whose catabolism is not directly affected by the enzymatic defect underlying the pathology. For this reason, we investigated this aspect in our NPA in vitro model. As shown in Fig. 3, SM-loaded NPA fibroblasts displayed an increase of phosphatidylcholine (PC) (2.5-fold vs unloaded NPA cells), phosphatidylethanolamine (PE) (eightfold vs unloaded NPA cells), ceramide (Cer) (1.3-fold vs unloaded NPA cells), glucosylceramide (GlcCer) (1.7-fold vs unloaded NPA cells), lactosylceramide (LacCer) (1.7-fold vs unloaded NPA cells), and globotriaosylceramide (Gb3) (twofold vs unloaded fibroblasts). In addition, ganglioside content also underwent a similar increase, being 2-, 1.5-, 4-, and 1.5-fold higher for GD1a, GD3, GM2, and GM3, respectively. Higher levels of cholesterol content were also observed (about 1.7-fold) in SM-loaded NPA fibroblasts with respect to unloaded NPA cells.

### SM Accumulation Induces TFEB Nuclear Translocation and Affects the Endolysosomal Compartment

**Fig. 4:**
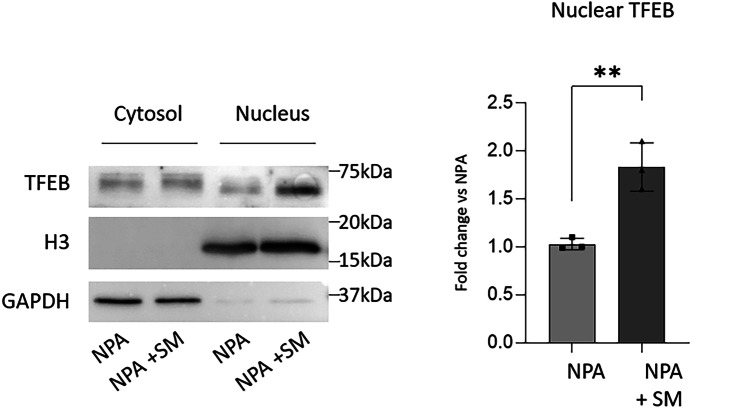
Effect of sphingomyelin accumulation on TFEB nuclear translocation in NPA fibroblasts. Immunoblotting analyses of the expression of the transcription factor EB (TFEB) in cytosolic or nuclear extracts of NPA fibroblasts loaded or not with 50 µM sphingomyelin (SM) for 30 days and subjected to nuclear and cytoplasmic extraction. Optical densities of the individual bands were quantified using NIH ImageJ and normalized to GAPDH as cytosolic marker or Histone H3 as nuclear marker. Data are expressed as fold change with respect to unloaded NPA fibroblasts and are the mean ± SEM of three different experiments. **p < 0.01. Two-tailed Student’s *t*-test vs unloaded NPA fibroblasts

Several lines of evidence indicate that the lysosomal accumulation of uncatabolized substrates, a typical feature of LSDs, triggers the nuclear translocation of TFEB (Samarani et al. 2018) which is involved in the regulation of several processes such as lysosomal biogenesis and exocytosis (Settembre and Medina 2015). We assessed TFEB nuclear translocation in NPA fibroblasts loaded with SM and we found that the nuclear counterpart of TFEB was about 1.7-fold higher with respect to unloaded NPA fibroblasts (Fig. 4).

**Fig. 5:**
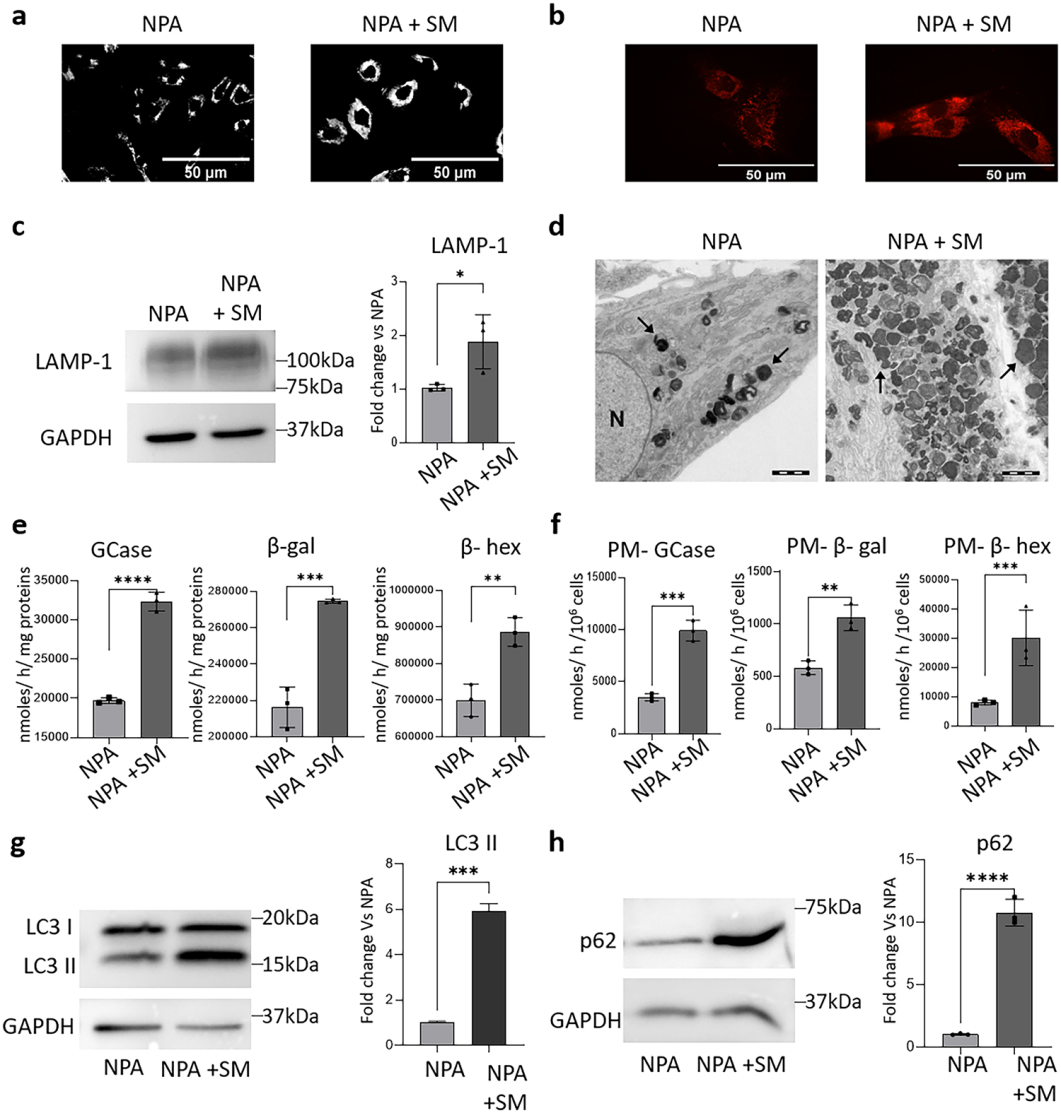
Effect of sphingomyelin accumulation on the lysosomal compartment in NPA fibroblasts. **a** Lysotracker DND-99 staining of NPA cells loaded or not with 50 µM SM for 30 days. Scale bar = 50 µm in both panels. **b** Expression of LAMP-1 in NPA fibroblasts loaded or not with 50 µM SM for 30 days, evaluated by immunofluorescence. Images were acquired at 200 × magnification. Scale bar = 50 µm in both panels. **c** Immunoblotting analyses of lysosomal-associated membrane protein (LAMP-1) in NPA fibroblasts loaded or not with 50 µM sphingomyelin (SM) for 30 days. **d** Representative electron microscopy images of lysosomes (arrows) in NPA fibroblasts loaded or not with 50 µM SM for 30 days. Scale bar = 2 µm in both panels. N = nucleus; **e** β-glucocerebrosidase (GCase), β-galactosidase (β-gal), and β-hexosaminidase (β-hex) activities were evaluated in NPA fibroblasts loaded or not with 50 µM SM for 30 days. **f** Specific enzymatic activity of plasma membrane–associated (PM) β-glucocerebrosidase, β-galactosidase, and β-hexosaminidase of NPA fibroblasts loaded or not with 50 µM SM for 30 days. Enzymatic activities are expressed as nmoles/h/cell mg of proteins (**e**) and nmoles/h/10.^6^ cells (**f**) and are the mean ± SEM of three experiments. Immunoblotting analyses of the expression of **g** LC3- II and **h** p62 in NPA cells loaded or not with 50 µM SM for 30 days. Optical densities of the individual bands were quantified using NIH ImageJ and normalized to GAPDH. Data are expressed as fold change with respect to unloaded NPA cells and are the mean ± SEM of three different experiments. *p < 0.05, **p < 0.01, ***p < 0.001; ****p < 0.0001; two-tailed Student’s *t*-test vs unloaded NPA cells

Next, we evaluated the effect of TFEB activation on lysosomal biogenesis by staining acidic organelles with LysotrackerRed DND-99 in live cells. As shown in Fig. 5a, SM-loaded NPA fibroblasts showed an enhanced fluorescent staining compared to unloaded NPA cells, indicating an increase in the relative volume of acidic intracellular compartments. In addition, by indirect immunofluorescence analyses against the lysosomal marker LAMP-1 on permeabilized cells, we observed a higher fluorescence intensity in SM-loaded NPA cells compared to the unloaded ones (Fig. 5b). An increased lysosomal biogenesis was also confirmed by immunoblotting analyses against LAMP-1, which showed almost doubled expression levels of LAMP-1 protein upon SM loading with respect to unloaded NPA cells (Fig. 5c). Electron transmission microscopy analyses revealed an increase not only in the number of lysosomes, but also in their volume upon SM loading (Fig. 5d). Moreover, in SM-loaded NPA fibroblasts, we observed an increase in the enzyme activities of several lysosomal glycohydrolases. In particular, the activity of β-glucocerebrosidase increased about 1.7-fold, while β-galactosidase and β-hexosaminidase activities increased about 1.2-fold with respect to unloaded cells (Fig. 5e).

Another effect of TFEB activation in response to lysosomal stress is the increase in lysosomes-PM fusion. This process leads to the association of lysosomal glycohydrolases with the external leaflet of the PM (Samarani et al. 2018). Interestingly, we observed an increase of about 2.5-fold in the activity of β-glucocerebrosidase, twofold in the activity of β-galactosidase, and 3.2-fold in the activity of β-hexosaminidase on the external leaflet of the PM of SM-loaded NPA fibroblasts compared to unloaded ones (Fig. 5f).

Lysosomes are essential players of the autophagic process, which serves for the degradation and recycling of cell components. To study the effect of lysosomal accumulation of SM on autophagy, we performed immunoblotting analyses against the autophagic marker LC3-II. As shown in Fig. 5g, it was about sixfold more expressed in SM-loaded NPA cells with respect to unloaded ones. In addition, we also found an increased expression of the autophagy substrate p62, about tenfold vs unloaded cells (Fig. 5h). Since upon SM loading, we found the increase of both LC3-II and p62 expression; a blockage of the autophagic flux seems to occur in SM-loaded NPA fibroblasts.

Overall, our results indicate that SM accumulation promotes TFEB nuclear translocation, which reflects in increased lysosomal biogenesis and autophagy impairment. In addition, the augmented activity of the PM-associated glycohydrolases suggests an activation of lysosomal exocytosis.

### Impact of Lysosomal SM Accumulation on the Cell Transcriptome

Since TFEB exerts its action on several genes and regulates several cell processes, to gain insight into the pathways primarily affected by SM accumulation, we performed RNA sequencing (RNAseq) experiments on NPA fibroblasts subjected or not to SM loading (3 replicates per condition, *N* = 6).

Pairwise correlation between untreated and SM-treated samples indicated high reproducibility and consistency of RNAseq replicates. Principal component analysis (PCA) on transcriptional data showed that samples separated mainly based on treatment, which explained 91% of the overall variance across replicates (Supplementary Fig. 1).

**Fig. 6:**
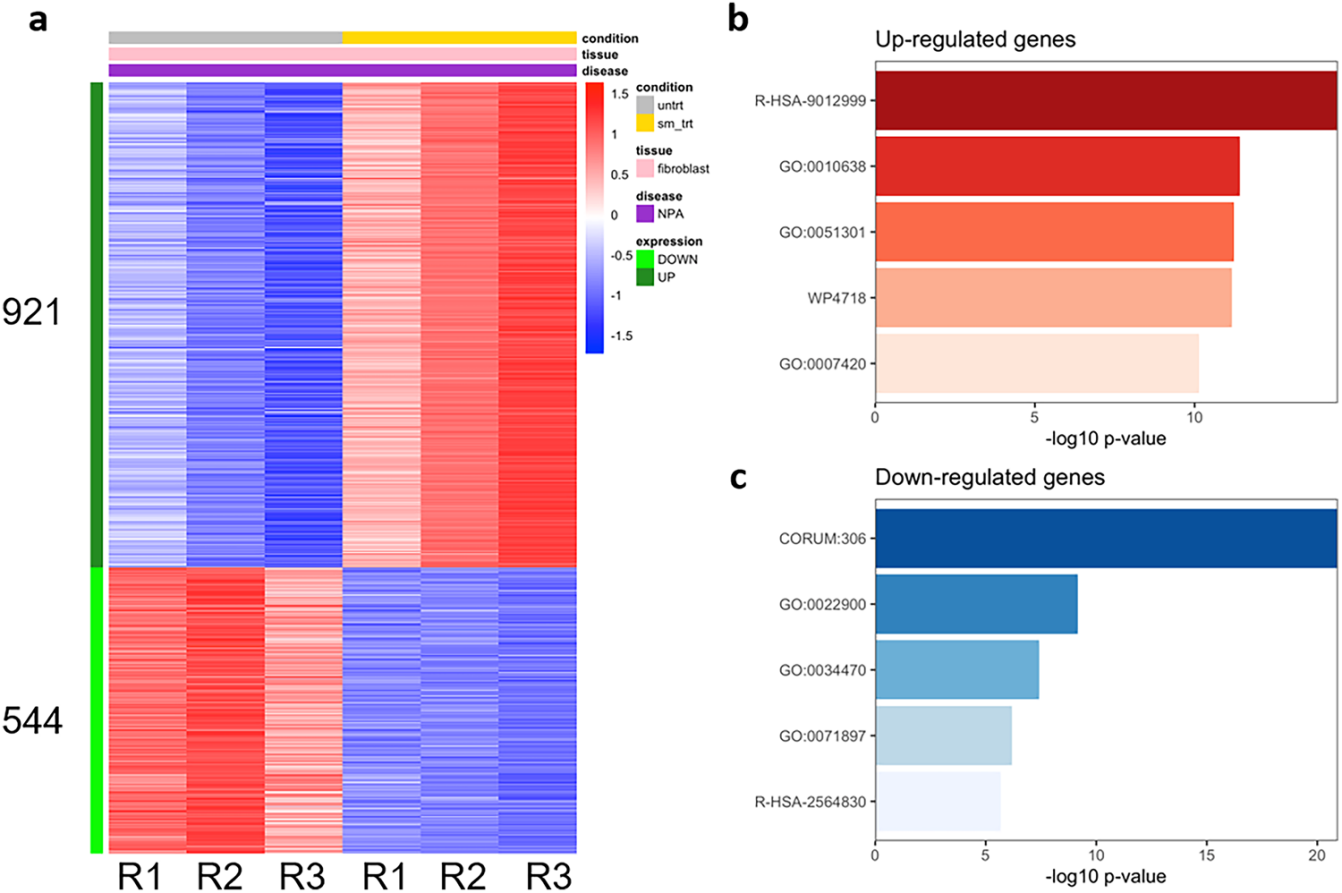
Effect of sphingomyelin accumulation on the transcriptional profile of NPA fibroblasts. **a** Heatmap showing differentially expressed genes (absolute log2FC > 1, adjusted *p*-value < 0.01) in NPA fibroblasts loaded or not with 50 µM sphingomyelin. **b**, **c** Gene ontology enrichment analysis showing the top 5 functional terms enriched among b sphingomyelin-induced (R-HSA-9012999 RHO GTPase cycle; GO:001,063 Positive regulation of organelle organization; GO:0,051,301 Cell division; WP4718 Cholesterol metabolism; GO:0,007,420: Brain development) and c sphingomyelin-repressed (CORUM:306 Ribosome cytoplasmic; GO:0,022,900 Electron transport chain; GO:0,034,470 ncRNA processing; GO:0,071,897 DNA biosynthetic process; R-HSA-2564830 Cytosolic iron-sulfur cluster assembly) genes

Differential gene expression analysis was then applied to identify transcriptional changes associated with the treatment: SM loading led to altered transcription of 1465 genes (921 upregulated and 544 downregulated) with absolute log2FC ≥ 1 and adjusted *p*-value ≤ 0.01 (Fig. 6a, Supplementary data). Among the upregulated genes, several were involved in cell division, lipid biosynthetic process, and cholesterol metabolism (GO:0,051,301 and WP4718, respectively, *p* < 1 × 10 − 10) (Fig. 6b, Supplementary Fig. 2); among downregulated ones, genes encoding for ribosomal proteins (CORUM:306, *p* < 1 × 10 − 20) and enzymes of the electron chain transport (GO:0,022,900, *p* < 1 × 10 − 9) were the most enriched (Fig. 6c, Supplementary Fig. 3).

### SM-Loaded NPA Fibroblasts Show Altered Mitochondrial Functions

The alterations in the regulation of cholesterol biosynthesis as well as its accumulation observed upon SM loading are typical features of other NP diseases, such as Niemann-Pick type C. In particular, alterations of the cholesterol content have been related to the onset of mitochondrial alterations (Yu et al. 2005; Torres et al. 2021).

**Fig. 7:**
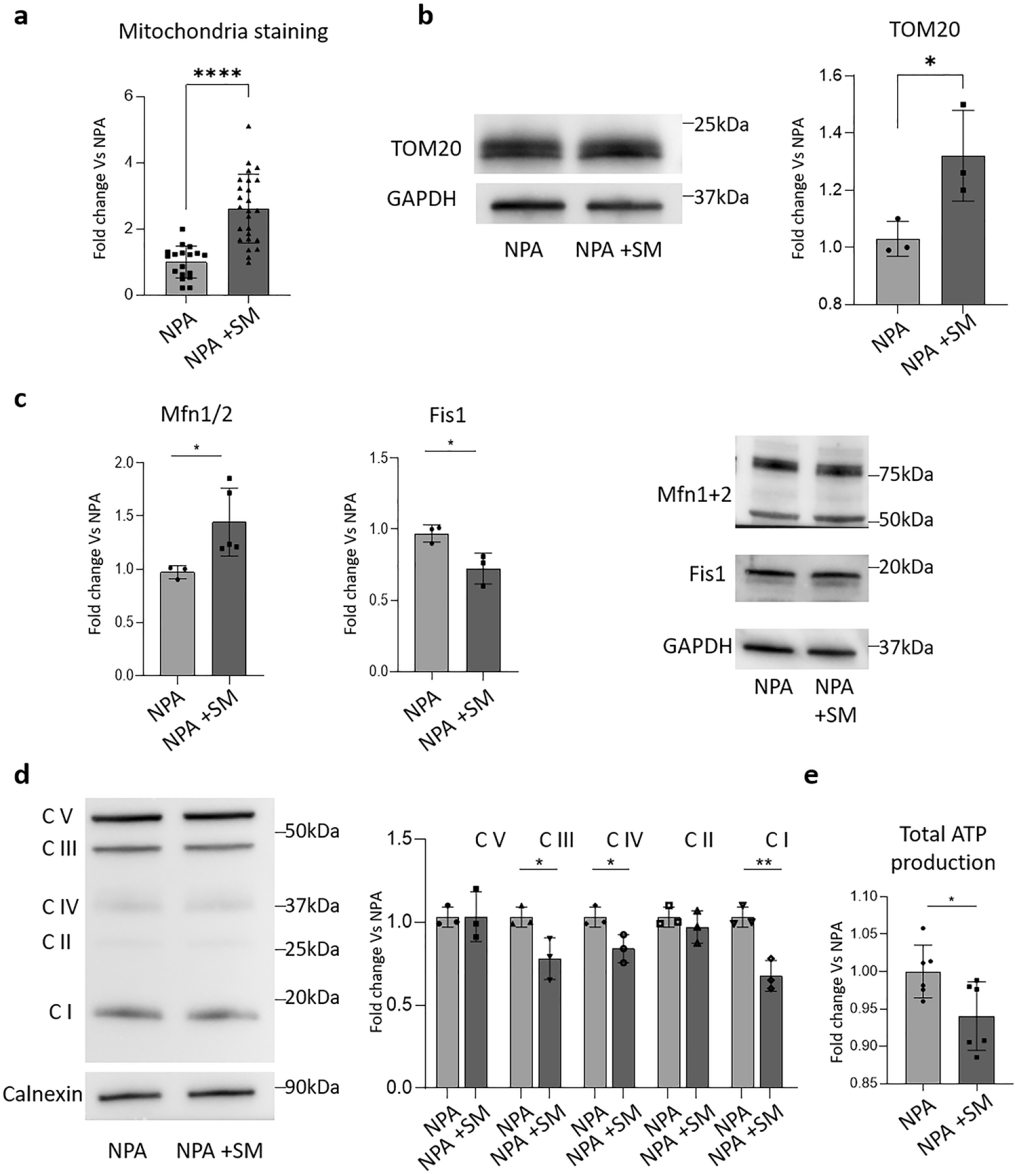
Effect of sphingomyelin accumulation on the mitochondrial compartment of NPA fibroblasts. **a** MitoTracker™ Deep Red FM staining of NPA cells loaded or not with 50 µM SM for 30 days. Fluorescence signal was quantified using NIH ImageJ. Immunoblotting analyses of the expression of **b** TOM20, **c** mitofusin 1 and 2, mitochondrial fission protein 1, and **d** complexes CV, CIV, CIII, CII, and CI of NPA fibroblasts loaded or not with SM for 30 days. Optical densities of the individual bands were quantified using NIH ImageJ and normalized to GAPDH or calnexin, respectively. **e** Total amount of cell ATP produced by NPA fibroblasts loaded or not with SM for 30 days. All data are expressed as fold change with respect to unloaded NPA cells and are the mean ± SEM of three different experiments. *p < 0.05; **p < 0.01; ***p < 0.001; ****p < 0.0001; two-tailed Student’s *t*-test vs NPA fibroblasts

We investigated the mitochondrial content of NPA cells, loaded or not with SM, by using both MitoTracker™ and immunoblotting analyses against the mitochondrial receptor TOM20. The staining with MitoTracker™ showed an increase in the mitochondria content of about 1.5-fold in SM-loaded NPA fibroblasts with respect to unloaded ones (Fig. 7a). Moreover, TOM20 protein expression increased by about 30% upon SM-loading in NPA fibroblasts (Fig. 7b).

To further evaluate the effect of SM loading on mitochondria homeostasis, we performed immunoblotting analysis of mitofusin 1 and 2 (Mfn) and of the mitochondria fission protein 1 (Fis1). Mfn1 and 2 expression resulted upregulated upon SM loading, suggesting an unbalance of mitochondrial dynamics towards the fusion process, whereas the expression of Fis1 resulted decreased, indicating a reduced mitochondrial fission (Fig. 7c).

Regarding the mitochondria functionality, we observed a reduced expression of mitochondria oxidative phosphorylation complexes. In particular, upon SM loading of NPA cells, complex I, III, and IV resulted in a 40%, 30%, and 20% reduced expression, respectively (Fig. 7d). These is in line also with RNAseq data showing a global downregulation of the genes involved in the electron transport chain. These data suggest a possible impairment in the mitochondrial respiratory activity and are also supported by a slight reduction in the total cell ATP production (Fig. 7e).

**Fig. 8:**
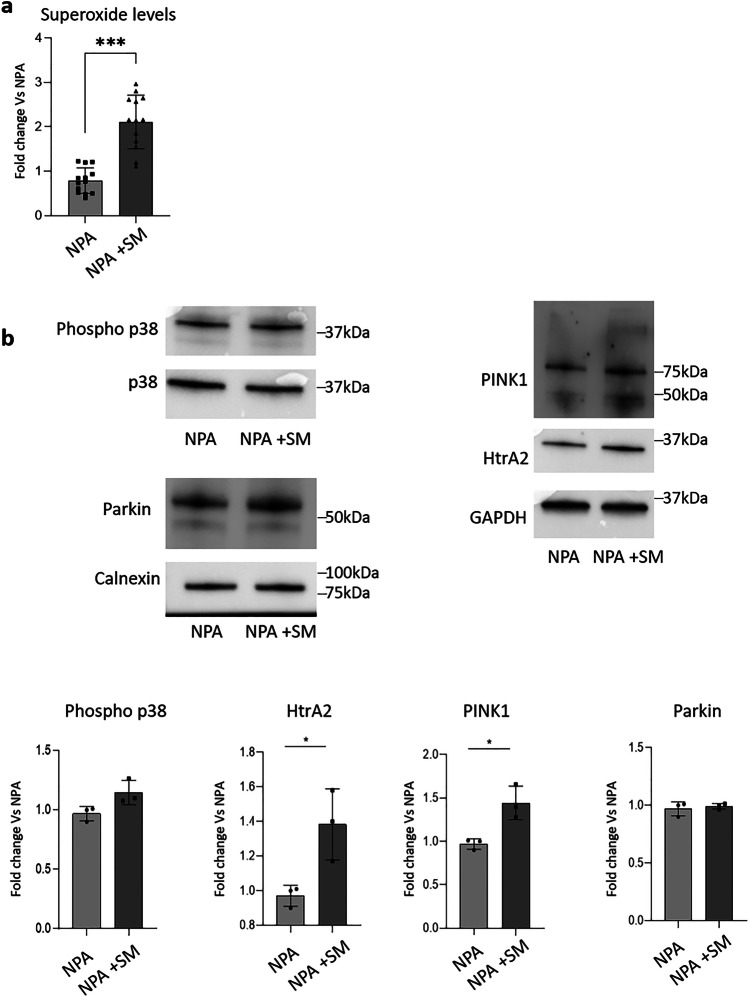
Effect of sphingomyelin accumulation on the mitochondrial quality control machinery. **a** MitoSOX™ Red staining of NPA cells loaded or not with 50 µM sphingomyelin (SM) for 30 days. Fluorescence signal was quantified using NIH ImageJ. **b** Immunoblotting analyses of the expression of the phosphorylated form of p38 (phospho p38), HtrA2, PINK1, and Parkin of NPA fibroblasts loaded or not with SM for 30 days. Optical densities of the individual bands were quantified using NIH ImageJ and normalized to p38 or GAPDH. All data are expressed as fold change with respect to unloaded NPA cells and are the mean ± SEM of at least three different experiments. *p < 0.05; ***p < 0.001; two-tailed Student’s *t*-test vs NPA fibroblasts

By using MitoSOX™ Red, we demonstrated that the aberrant accumulation of SM in NPA cells loaded with exogenous SM induced a twofold increase in the production of superoxide species compared to untreated cells (Fig. 8a).

Oxidative stress can be detrimental to cell homeostasis and cells try to counteract the damage by activating defense mechanisms such as mitochondrial molecular quality control and organellar quality control, i.e., mitophagy. We evaluated the expression of proteins involved in both pathways. Upon SM loading, the content of PINK1 and HtrA2 proteins resulted increased and was accompanied by an increasing trend of the phosphorylated and active p38 stress kinase. SM loading of NPA fibroblasts did not lead to alterations of Parkin, one of the main actors of the organellar quality control machinery (Fig. 8b).

### The Brains of ASMKO Mice Show an Increased TFEB Nuclear Translocation and the Impairment of the Lysosomal Compartment

The ASMKO mouse model is a widely used in vivo model that recapitulates the NPA pathology. Interestingly, starting from 3 months of age, ASMKO mice begin to show the onset of a neurodegenerative phenotype that progressively worsens leading to death. It has been described in literature that the onset of the neuronal damage correlates with the levels of the accumulated SM. In particular, at 7 months of age, these mice show a severe neurodegenerative phenotype that correlates with a significant accumulation of SM in the brain, reaching a ninefold higher SM content with respect to that found in the brains of wild type (WT) mice (Horinouchi et al. 1995). Since we found a similar increase of SM content in our NPA in vitro model (Fig. 2a), we decided to validate the involvement of some molecular pathway described in SM-loaded NPA fibroblasts also in the *postmortem* brains obtained from 7-month-old ASMKO mice.

**Fig. 9:**
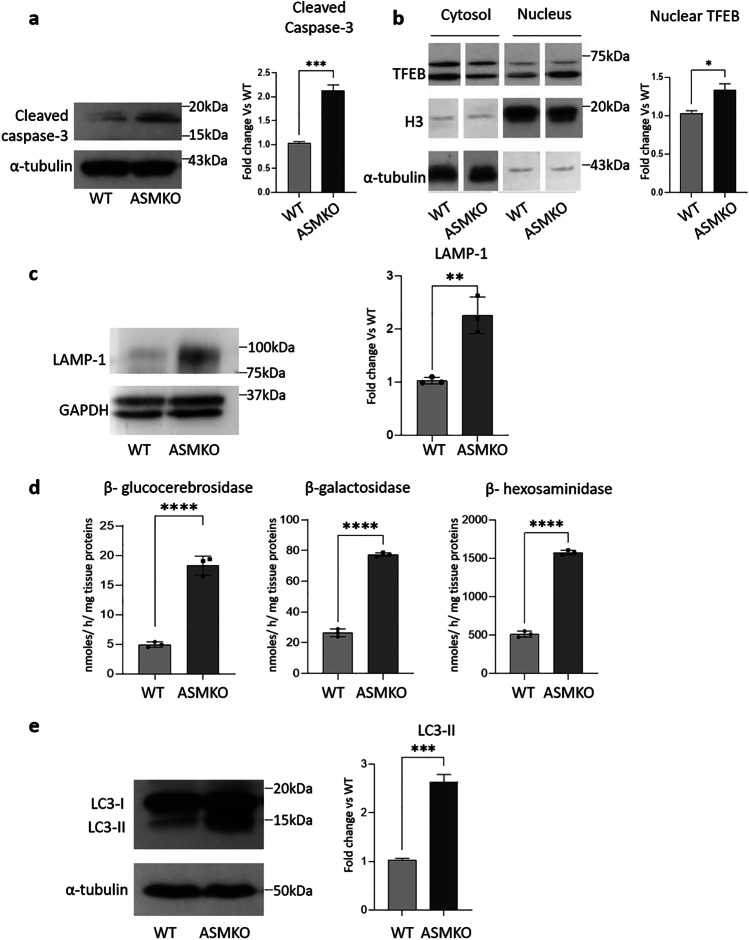
Effect of sphingomyelin accumulation on cell damage, on the lysosomal compartment, and autophagy of ASMKO mouse cerebellum. Immunoblotting analysis of the expression of **a** cleaved caspase-3, **b** the transcription factor EB (TFEB) in cytosolic and nuclear extracts, **c** the lysosomal marker LAMP-1, and **d** the autophagy marker LC3II in the cerebellum of 7-month-old wild type (WT) mouse and mouse knockout for the acidic sphingomyelinase enzyme (ASMKO). Optical densities of the individual bands were quantified using NIH ImageJ and normalized to α-tubulin or GAPDH. Data are expressed as fold change with respect to WT mouse cerebellum and are the mean ± SEM of three different experiments. **p < 0.01, ***p < 0.001; two-tailed Student’s *t*-test vs WT mouse cerebellum. e β-glucocerebrosidase, β-galactosidase, and β-hexosaminidase activities were evaluated in the cerebellum of WT and ASMKO mice at 7 months. Enzyme activities are expressed as nmoles of formed product/hour/mg tissue proteins. Data are the mean ± SEM of three different experiments, ****p < 0.0001; two-tailed Student’s *t*-test vs WT mouse cerebellum

First, we assessed whether the SM accumulation was sufficient to induce cell death by evaluating the expression of the cleaved form of the caspase 3. As shown in Fig. 8, its expression levels increased of about twofold in the brains of ASMKO mice compared to WT brains. Then, we found 25% increase of nuclear TFEB in ASMKO brains with respect to WT ones (Fig. 9). Furthermore, we observed a 2.2-fold increased expression of LAMP-1 in ASMKO brains with respect to WT brains (Fig. 10a). In addition, the brains of ASMKO mice showed an increased activity of the main lysosomal glycohydrolases. In particular, β-glucocerebrosidase activity was 3.5-fold higher, and β-galactosidase and β-hexosaminidase activities were about threefold higher in ASMKO brains with respect to WT brains (Fig. 10b). Finally, we evaluated the effect of the lack of ASM on the autophagy flux by performing immunoblotting analyses on the autophagy marker LC3-II. We observed an increase of about 2.6-fold in the LC3-II expression levels in ASMKO brains compared to the WT ones (Fig. 11). Taken together, our experiments performed on ASMKO mice brains indicate the involvement of the lysosomal impairment and the autophagy blockage in the onset of cell death, supporting the findings obtained in our previously described NPA in vitro model.

## Discussion

NPA is a LSD caused by mutations in the *SMPD1* gene, leading to decreased activity of the enzyme ASM, which results in lysosomal accumulation of SM. NPA affects multiple organs and determines a severe impairment of the central nervous system. Although the scientific community has deeply investigated NPA pathology in the last decades, the molecular mechanisms linking SM accumulation with the onset of cell damage and in particular with neurodegeneration are still not fully understood.

The broadly used model for the study of NPA disease is the ASMKO mouse, which is characterized by *SMPD1* gene knock-out and the consequent deficiency of ASM activity. The studies performed with this mouse model revealed a clear correlation between the levels of the accumulated SM and the onset of cell damage. Interestingly, these studies supported the idea that neurodegeneration is induced only if SM lysosomal accumulation exceeds a threshold amount. For instance, this could explain why the neurons isolated from ASMKO pups show a milder neurodegenerative phenotype. Another widely used in vitro model for the study of NPA disease is represented by skin fibroblasts isolated from NPA patients. These cell lines are stable and can be maintained in culture for long periods. Unfortunately, even if they show a low ASM residual activity, the entity of the accumulated SM is not related to an altered cell viability (Fig. 2c).

For this reason, to study the effect of the aberrant lysosomal accumulation of SM on cell homeostasis, we developed a strategy to force the accumulation of SM in NPA fibroblasts consisting in feeding cells with exogenous SM for 30 days. This condition allows to induce an increase in the SM content of tenfold higher compared to WT fibroblasts.

The biochemical characterization of this model highlights a general failure of the lysosomal compartment, together with an impairment of the autophagic flux and cell death. In particular, the autophagolysosomes of cells overloaded with SM were not able to catabolize the internalized substrates, thus leading to the accumulation of macromolecules such as GlcCer, LacCer, Gb3, PC, PE, gangliosides, ceramide, and cholesterol. The secondary accumulation of lipid species is a typical feature described among LSDs (Hůlková et al. 2005; Walkley and Vanier 2009) and although it is still under debate, increasing evidence indicates that this accumulation is caused by a general impairment of the catabolic activity of lysosomes.

Lysosomal stress due to the accumulation of uncatabolized substrates is also responsible for the establishment of a pathogenic lysosome-PM axis that determines the onset of cell damage (Gabandé-Rodríguez et al. 2014). The central player of this process is TFEB that, upon lysosomal impairment, translocates into the nucleus and promotes two events: lysosomal biogenesis and lysosomal fusion to the plasma membrane (Settembre and Medina 2015). In particular, during the fusion of the lysosomal membranes to the PM, lysosomal exocytosis leads to the release of the lysosomal content in the extracellular milieu and alters the structure of the PM. In an artificial model of lysosomal accumulation, the association of the lysosomal hydrolases with the external leaflet of the PM promotes the aberrant catabolism of the SLs which in turn leads to the onset of cell damage (Samarani et al. 2018). Also, in our NPA model, we found that the aberrant accumulation of SM induces lysosomal biogenesis and a marked increase in the activity of the PM-associated glycohydrolases, as a possible consequence of an augmented lysosomal exocytosis. Interestingly, we found an increased TFEB nuclear translocation also in the brain of ASMKO mice at the neurodegenerative stage, then likely inducing similar secondary effects as observed in the NPA in vitro model. Moreover, we also observed an aberrant production of lysosomes and the impairment of the autophagic flux. The data obtained in ASMKO brains resemble those obtained in our new NPA in vitro model, suggesting the involvement of SM accumulation in activating the lysosome-PM axis responsible for the onset of cell death also in vivo.

Interestingly, we found that the aberrant accumulation of SM modulates the expression of more than 1400 genes with an important effect on the cell homeostasis. In particular, enrichment analyses pointed to electron transport chain machinery and cholesterol biosynthesis. The accumulation of cholesterol observed in our NPA in vitro model is of particular interest since it was found to be responsible for the onset of mitochondrial damage in other LSDs (Yu et al. 2005). Indeed, the mitochondrial membrane, compared to other subcellular membranes, is particularly sensitive to cholesterol changes, which can affect the biophysical and functional characteristics of these organelles (Elustondo et al. 2017). Of note, the SM accumulation induced in our NPA cell model seems also to affect the mitochondrial compartment, thus contributing to the impairment of cell homeostasis. In particular, an overall increase in the number of mitochondria per cell is observed, even if we found an unbalance in other two fundamental processes for mitochondria homeostasis, such as the fusion and the fission. The onset of mitochondrial damage was also supported by the decreased expression of complex I, III, and IV subunits (in turn suggesting an impairment in mitochondrial function). In addition, we observed a slight reduction in the ATP content. This could be due to the fact that the major ATP production in fibroblasts mainly relies on oxidative metabolism, thus explaining why alterations in the mitochondrial complexes would affect the total ATP amount. On the other hand, deficits in mitochondrial activity have been reported to trigger mitochondria biogenesis as a compensatory mechanism, generating a pathogenic loop (Valero 2014; Roca-Agujetas et al. 2021).

Furthermore, the control of mitochondrial homeostasis is essential to maintain cell physiology and relies mainly on molecular and organellar quality control mechanisms. In particular, the quality control machinery seems to be activated in our NPA model upon SM loading, and, in particular, a higher proteolytic activity of HtrA2 could contribute to the control of mitochondrial damage. Furthermore, the lysosomal impairment could prevent the degradation of defective mitochondria, leading to their accumulation and to the exacerbation of oxidative stress that contributes to the observed cell damage (Rajman et al. 2018; Schöndorf et al. 2018). The data obtained on our NPA model shed light on the necessity to overcome a threshold of lysosomal accumulation of undegraded material in order to evocate a cell damage. In particular, this aspect is cell-related and needs to be considered for all the studies that want to address the etiopathology not only of NPA, but also of the other lysosomal storage disorders. Indeed, even if the NPA fibroblasts accumulate sphingomyelin, the levels are not sufficient to induce cell damage. For this reason, we think that our model could be considered for the future studies aimed to dissect the different molecular pathways compromised by the aberrant accumulation of sphingomyelin. Taken together, our study suggests that the massive accumulation of SM activates an aberrant lysosome-PM axis and impairs the mitochondrial function. These events could be related to the onset of cell death, opening a new scenario for the study of the etiopathological mechanism of NPA.

## Supplementary Information

Below is the link to the electronic supplementary material.

Supplementary file1 (DOCX 652 KB)
