# Supplementary material for: Massive Accumulation of Sphingomyelin Affects the Lysosomal and Mitochondria Compartments and Promotes Apoptosis in Niemann-Pick Disease Type A
Source: J Mol Neurosci. 2022 Jun 21;72(7):1482–99. doi: 10.1007/s12031-022-02036-4 (PMC9293875; doi:10.1007/s12031-022-02036-4)

**Massive accumulation of sphingomyelin affects the lysosomal and mitochondria compartments and promotes apoptosis in Niemann- Pick disease type A**

*Journal of Molecular Neuroscience*

Emma Veronica Carsana^1^, Giulia Lunghi^1^, Simona Prioni^1^, Laura Mauri^1^, Nicoletta Loberto^1^, Alessandro Prinetti^1^, Fabio A. Zucca^2^, Rosaria Bassi^1^, Sandro Sonnino^1^, Elena Chiricozzi^1^, Stefano Duga^3,4,†^, Letizia Straniero^3,4^, Rosanna Asselta^3,4^, Giulia Soldà^3,4^, Maura Samarani^5 Ϯ^, Massimo Aureli^1Ϯ^

^1^Department of Medical Biotechnology and Translational Medicine, University of Milan, Milan, Italy

^2^Institute of Biomedical Technologies, National Research Council of Italy, Segrate, Italy

^3^Department of Biomedical Sciences, Humanitas University, Via Rita Levi Montalcini 4, 20090 Pieve Emanuele, Milan, Italy

^4^Humanitas Clinical and Research Center, IRCCS, Via Manzoni 56, 20072 Rozzano, Milan, Italy

^5^Department of Cell Biology and Infection, Institut Pasteur, Paris, France

^Ϯ^ These authors share the senior position

Corresponding author:

Massimo Aureli, Department of Medical Biotechnology and Translational Medicine, University of Milan, Milan, Italy, Tel: 0250330364; massimo.aureli@unimi.it

**Supplementary Figure 1. RNAseq data analysis of NPA fibroblasts**

Pairwise correlation between samples of NPA fibroblasts loaded (**b**) or not (**a**) with 50 µM sphingomyelin (SM). In the lower panels, scatterplots show the reproducibility of RNAseq biological and technical replicates. In the upper panels, the corresponding Pearson’s correlation coefficients (rho) and significance (***p<0.001) are reported. **c** Principal component (PC) analysis of RNA expression of NPA fibroblasts, loaded (red) or not (blue) with SM. X and Y axes represent the first and the second PC, respectively. **d** Volcano plot showing differentially expressed genes (absolute log2FC > 1, adjusted p-value < 0.01) up-regulated (right) or down-regulated (left) in SM-loaded vs unloaded NPA fibroblasts.

**Supplementary Figure 2. Effect of sphingomyelin accumulation on the transcriptional profile of genes involved in the cholesterol biosynthesis in NPA fibroblasts**

The list of 47 genes belonging to the WikiPathways WP4718, Cholesterol metabolism with Bloch and Kandutsch-Russell pathways, was downloaded from the GSEA website (<https://www.gsea-msigdb.org/gsea/msigdb/cards/WP_CHOLESTEROL_METABOLISM_WITH_BLOCH_AND_KANDUTSCHRUSSELL_PATHWAYS.html>)

**a** Volcano plot showing a generalized upregulation of genes involved in cholesterol metabolism upon sphingomyelin accumulation. For each gene, the fold change is plotted on the horizontal axis and the FDR-adjusted p-value on the vertical axis. The red dotted lines represent the thresholds of fold change (>|1|) and p-value (<0.01) used to select the differentially expressed genes.

**b** Heatmap showing the expression of genes involved in cholesterol metabolism in NPA fibroblasts loaded or not with 50 µM sphingomyelin.

**Supplementary Figure 3. Effect of sphingomyelin accumulation on the transcriptional profile of genes involved in the electron transport chain in NPA fibroblasts.**

The list of 106 genes belonging to the WikiPathways WP111, Electron transport chain: OXPHOS system in mitochondria was downloaded from the GSEA website (<https://www.gsea-msigdb.org/gsea/msigdb/cards/WP_ELECTRON_TRANSPORT_CHAIN_OXPHOS_SYSTEM_IN_MITOCHONDRIA.html>)

**a** Volcano plot showing a generalized downregulation of genes belonging to the mitochondrial electron transport chain upon sphingomyelin accumulation. For each gene, the fold change is plotted on the horizontal axis and the FDR-adjusted p-value on the vertical axis. The red dotted lines represent the thresholds of fold change (>|1|) and p-value (<0.01) used to select the differentially expressed genes. Most genes are significantly downregulated, although the fold change is modest.

**b**Heatmap showing the expression of genes belonging to the mitochondrial electron transport chain in NPA fibroblasts loaded or not with 50 µM sphingomyelin.

Supplementary Figure 1
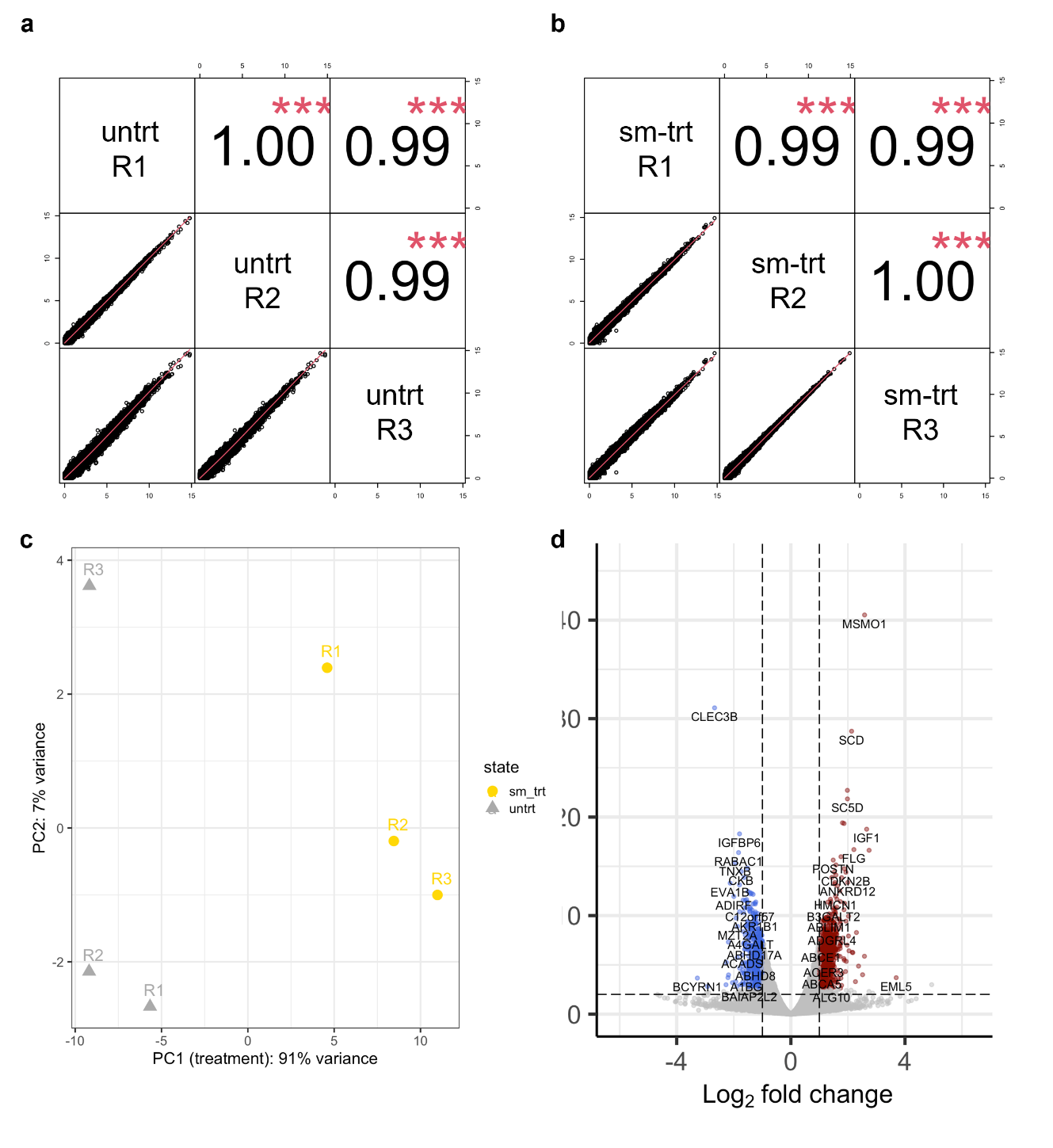


Supplementary Figure 2
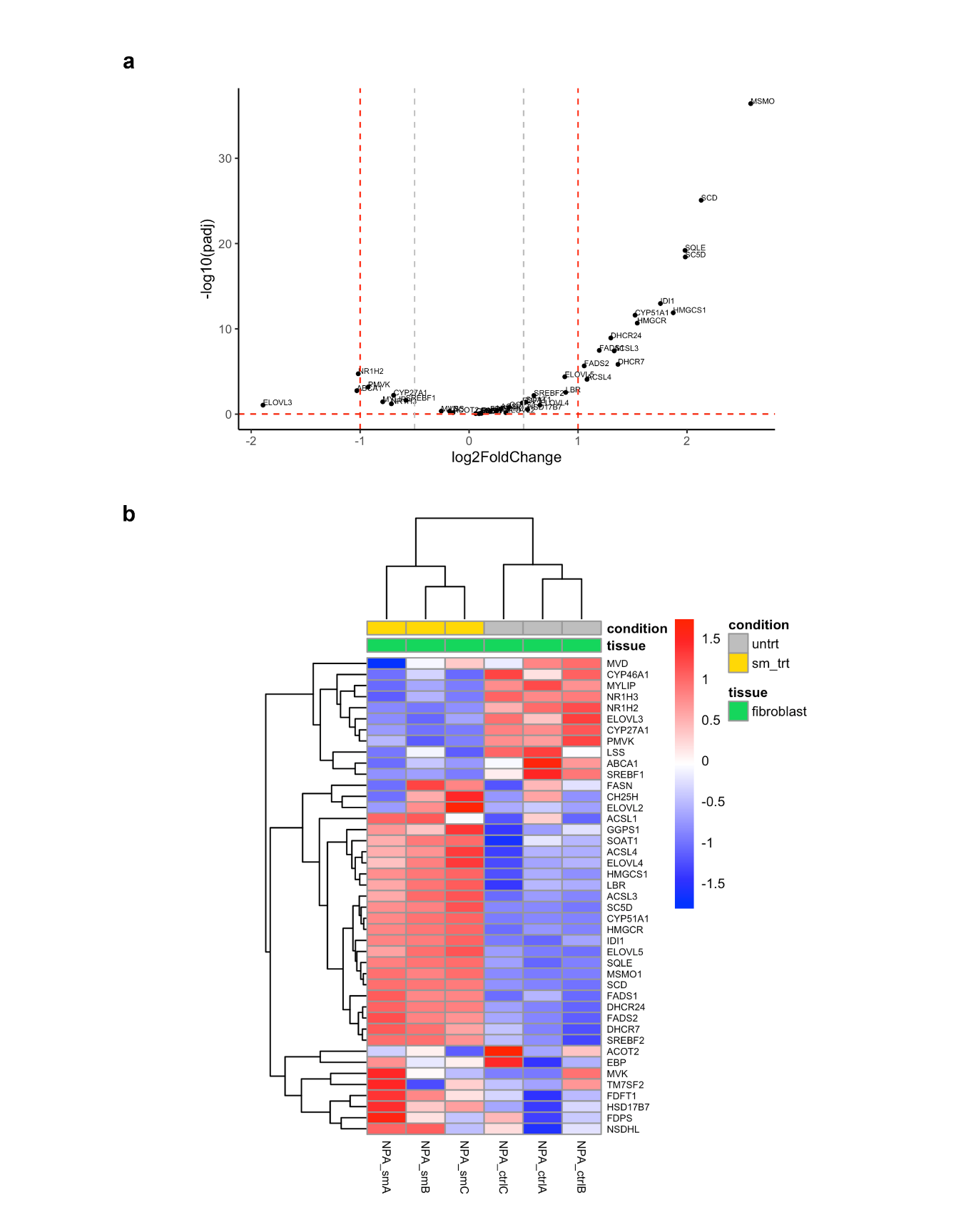


Supplementary Figure 3
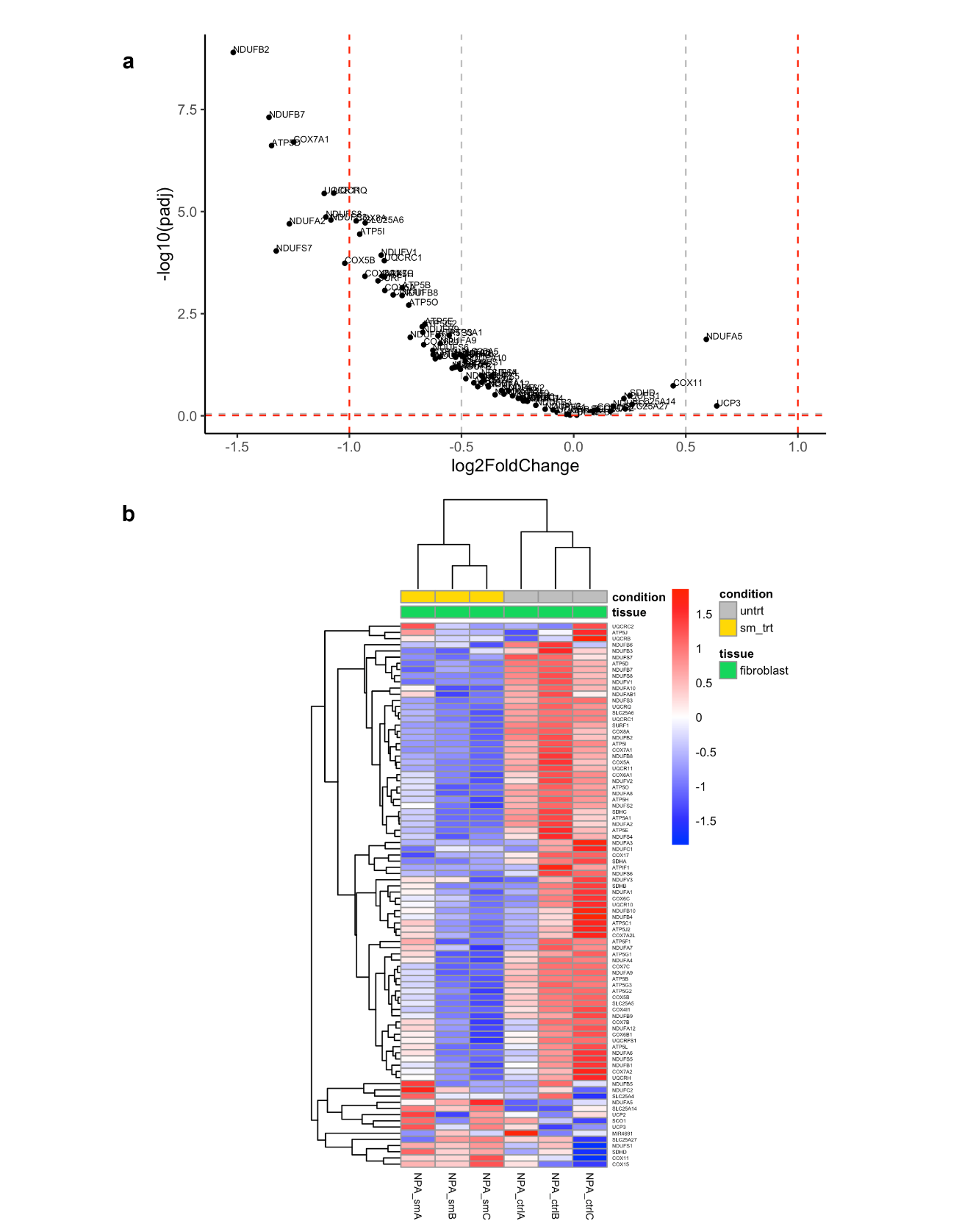

Supplement: Supplementary file 1 — Supplementary file1 (DOCX 652 KB) [file 12031_2022_2036_MOESM1_ESM.docx]
